# Correction to “Barna T, Szucs KF, Schaffer A, Mirdamadi M, Hajagos‐Toth J, Gaspar R. Combined uterorelaxant effect of magnesium sulfate and terbutaline: Studies on late pregnant rat uteri in vitro and in vivo. Acta Obstet Gynecol Scand. 2023; 102(4): 457–464. PMID: 36808376; PMCID: PMC10008284”

**DOI:** 10.1111/aogs.14809

**Published:** 2024-02-10

In sections 2.4. and 2.5. of the originally published version of the article, the information provided about the manufacturer and distributor of the bipolar disk electrode SEN‐15‐2 was incorrect. The correct name of the company manufacturing and distributing the device is MSB‐MET Ltd, Balatonfüred, Hungary.

We apologize for this error.

